# Investigating the Mechanical Characteristics and Fracture Morphologies of Basalt Fiber Concrete: Insights from Uniaxial Compression Tests and Meshless Numerical Simulations

**DOI:** 10.3390/ma17215258

**Published:** 2024-10-29

**Authors:** Chuan Zhao, Guoxin Jiang, Junli Guo, Shuyang Yu, Zelong Ma, Chunyi Zhuang, Youbin Lei, Zilin Liang

**Affiliations:** 1 Sichuan Academy of Water Conservancy, Chengdu 610072, China; zhaochuanvip@163.com (C.Z.); jianggx81@163.com (G.J.); guojunli99988@163.com (J.G.); mazelong1234562024@163.com (Z.M.); zhuangchunyi@126.com (C.Z.); 2 School of Transportation and Civil Engineering, Nantong University, Nantong 226019, China; 19517111903@163.com; 3 Dujiangyan Irrigation Project Hangshi Zhida Industrial Co., Chengdu 610072, China; 13880981205@163.com

**Keywords:** basalt fiber-reinforced concrete, uniaxial compression, fracture mode, meshless method, numerical simulation

## Abstract

To explore the mechanical properties and fracture modes of basalt fiber-reinforced concrete, single-doped and hybrid-doped basalt fiber-reinforced concrete was prepared, and uniaxial failure tests under different basalt fiber-reinforced concrete contents were carried out. At the same time, the smooth kernel function in the traditional SPH method was improved, and the basalt fiber random generation algorithm was embedded in the SPH program to realize the simulation of the progressive failure of basalt fiber-reinforced concrete. The results show that under the circumstance with no basalt fiber, the specimen final failure mode is damage on the upper and lower surface, as well as the side edge, while the interior of the specimen center is basically intact, indicating that there is an obvious stress concentration phenomenon on the upper and lower surface when the specimen is compressed. Under the circumstance with basalt fiber, longitudinal cracks begin to appear inside the specimen. With the increase in the content, the crack location gradually develops from the edge to the middle, and the crack number gradually increases. This indicates that appropriately increasing the fiber content in concrete may improve the stress state of concrete, change the eccentric compression to axial compression, and indirectly increase the compressive strength of concrete. The numerical simulation results are consistent with the test results, verifying the rationality of the numerical simulation algorithm. For the concrete model without the basalt fiber, shear cracks are generated around the model. For the concrete model with basalt fiber, in addition to shear cracks, the tensile cracks generated at the basalt fiber inside the model eventually lead to the splitting failure of the model. The strength of concrete samples with basalt content of 0.1%, 0.2%, and 0.3% is increased by 1.69%, 5.10%, and 4.31%, respectively, compared to the concrete sample without basalt fiber. It can be seen that with the increase in the content of single-doped basalt fiber, the concrete strength is improved to a certain extent, but the improvement degree is not high; For hybrid-doped basalt fiber-reinforced concrete, the strength of concrete samples with basalt content of 0.1%, 0.2%, and 0.3% is increased by 14.51%, 15.02%, and 30.31%, respectively, compared to the concrete sample without basalt fiber. Therefore, compared with the single-doped basalt fiber process, hybrid doping is easier to improve the strength of concrete.

## 1. Introduction

**Figure 1:**
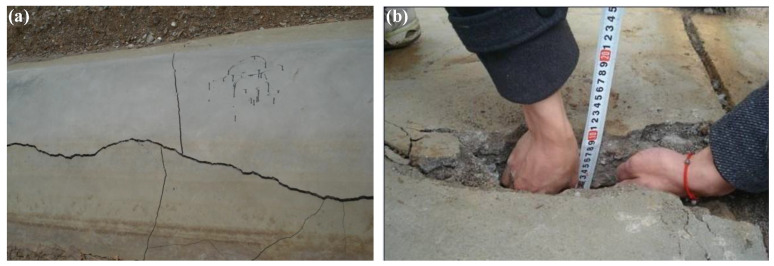
Concrete cracking morphology of one water conservancy project channel in western China. (**a**) Damage location; (**b**) maximum crack depth.

In recent years, with the rapid development of China’s water conservancy infrastructure construction, how to improve the safety and durability of hydraulic structure buildings has gradually become a hot and difficult point in engineering construction [1]. Under unfavorable service environment conditions such as large temperature differences between day and night, high-frequency freeze–thaw, strong ultraviolet radiation, and dry–wet cycles, hydraulic concrete is extremely prone to deterioration phenomena, such as freeze–thaw damage, scour and wear, cracking and leakage, and surface spalling [2,3]. The authors studied the construction situation of one water conservancy project at an altitude of 3500 m in western China. They found that the channels just poured last year and the installed “U”-shaped channels had different degrees of deformation and damage after one winter. The maximum crack depth reached 100 mm and the seepage was serious, as shown in Figure 1. Therefore, concrete deterioration can lead to failure of the structural function of hydraulic structures, affect the safe operation of equipment, and reduce the comprehensive benefits of hydropower stations. The main reason for the insufficient durability of concrete is that traditional concrete has high compressive strength but low tensile strength, and has poor ability to adapt to tensile deformation or uneven deformation [4,5]. In view of this, considering adding high-performance fiber materials such as basalt fiber to traditional concrete [6], enhancing the durability index of traditional concrete, improving the toughness of concrete structure, optimizing the mix proportion design and preparation process of high-performance basalt fiber-reinforced concrete, and prolonging the service life of hydraulic structures will be of great significance for improving the energy efficiency of water conservancy project construction.

Research on the mechanical and fracture characteristics of basalt fiber-reinforced concrete mainly focuses on three aspects: experimental research, theoretical research, and numerical simulation research. Experimental research is the most intuitive means to reflect the mechanical properties of basalt fiber-reinforced concrete and reveal its fracture morphology. For example, Huo et al. [7] carried out mechanical property and impermeability tests on concrete with basalt chopped fibers of different lengths. By comparing the characteristic values and variation laws of each group of fibers, the optimal length of the fibers was determined. Zhang et al. [8] conducted experimental research on the dynamic splitting tensile properties of polypropylene fiber and basalt fiber-reinforced concrete and analyzed the action mechanism of polypropylene fiber and basalt fiber. Zhang et al. [9] investigated the long-term durability of basalt fiber-reinforced geopolymer concrete under dry–wet cycles and immersion treatment in marine conditions. Fu et al. [10] determined the sulfate concentration in the field gypsum rock stratum environment. Then, through mechanical tests, the changes in mechanical properties of basalt fiber concrete (BFC) before and after sulfate erosion were analyzed. Wang et al. [11] prepared three repair materials, beryllium copper concrete, geopolymer mortar, and basalt fiber-reinforced geopolymer mortar, on the rough surface of old base materials and exposed them for 1 h under high-temperature conditions. The interfacial bonding strength was tested through shear tests. However, experimental research can only obtain the stress and strain data and the final sample failure morphology of basalt fiber-reinforced concrete, but it is difficult to intuitively reveal its internal fracture mechanisms. Theoretical research is based on the results of experimental research and summarizes, as well as refines, concrete fracture criteria or damage evolution models. Concrete fracture mechanics originated from the fracture mechanics theory proposed by Griffith [12]. Obreimoff [13] verified the universality of Griffith’s theory through mica splitting tests in 1930. Orowan [14] studied the influence of material plasticity on crack propagation in 1942 and revised Griffith’s theory and proposed the Griffith formula considering plastic work. Sneddon [15] proved the singularity of the stress component at the crack tip with *r*^−1/2^ in terms of mechanical prospect in 1946. Rice [16] proposed the concept of the J-integral in 1968. Subsequently, based on classical fracture mechanics theory, scholars have developed and improved concrete fracture mechanics. For example, Bažant et al. [17] proposed an “m index” for concrete fracture mechanics. Zhao [18] analyzed the fracture law of gravity dams using linear elastic fracture mechanics theory. Sheng et al. [19] studied the splitting mechanism and fracture criterion of hydraulic tunnels under high water head using fracture mechanics principles. However, theoretical research can only obtain analytical solutions under simple geometric and boundary conditions. For complex situations, such as the complex structure of basalt fiber-reinforced concrete, it is difficult to produce accurate answers using traditional concrete fracture mechanics.

Numerical simulation can intuitively show the fracture mechanisms of basalt fiber-reinforced concrete. The finite element method is one of the earliest numerical methods for simulating the concrete fracture. For example, Min et al. [20] proposed a numerical simulation method for irregular cross-scale cracks in mass concrete structures based on a node projection strategy using the finite element method. Qu et al. [21] used the cohesive zone model in the finite element method to study the seismic cracking evolution process of concrete slabs. Cao et al. [22] used the three-dimensional finite element method to simulate the temperature and thermal stress distribution of concrete overflow dams during construction. However, in dealing with discontinuous problems such as crack propagation, the finite element method needs to constantly change the mesh division around the crack, which consumes a large amount of computing resources. Subsequently, people proposed the discrete element method to simulate the failure process of concrete. For example, Nitka et al. [23] used the classical particle discrete element method to numerically simulate the complex fracture process of concrete beams under quasi-static three-point bending. Wang et al. [24] simulated the cracking process of asphalt concrete under low-temperature conditions using the SCB loading mode, based on the discrete element method. Zhou et al. [25] used the discrete element method to construct a three-dimensional five-phase mesoscale model composed of mortar, coarse aggregate, polypropylene fiber, steel fiber, and a cross-section transition zone, and carried out stress-strain simulation and final fracture morphology simulation under compression conditions. However, the discrete element method has many mesoscopic parameters without practical physical meaning. Before simulation, it needs to go through complex parameter calibration, and the values of mesoscopic parameters mostly depend on experience. In recent years, new numerical simulation methods such as peridynamics [26,27] and the numerical manifold method [28] all have good applications in concrete fracture simulation, but there are still some shortcomings. For example, in the bond-based peridynamics method, Poisson’s ratio is a constant, which is inconsistent with the real situation. The crack tip of the numerical manifold method can only fall on the mesh, and so on.

Smoothed particle hydrodynamics (SPH) is a meshless method. This method discretizes the computational domain into regularly distributed particles and transmits calculation parameters through the control equations obtained by discretizing strictly derived partial differential equations. Compared to the finite element method, SPH overcomes the problem of mesh redivision in dealing with large deformation and discontinuous problems. Compared to the discrete element method, the calculation parameters in SPH are strictly derived from partial differential equations and have clear physical meanings, without the need for complex parameter calibrations. SPH was initially used to simulate astrophysical problems such as planetary collisions, etc. [29,30], and then it has been widely used in complex fluid dynamics problems [31,32]. Due to the unique advantages of SPH in handling large deformations and discontinuities, it can handle rock crack propagation problems very conveniently. At present, SPH has many applications in rock fracture simulation, such as the GPD method proposed by Zhou et al. [33,34,35,36,37,38,39,40,41]. However, the application of SPH in simulating the concrete fracture process is still relatively rare.

Based on the deficiencies of previous studies, this paper considers adding high-performance fiber materials, such as basalt fiber, to traditional concrete to enhance the durability performance indicators such as tensile strength and frost resistance of traditional concrete and improve the toughness of concrete structures. In the experiment, two schemes of single doping and hybrid doping are adopted to obtain the optimal ratio scheme through comparison. The traditional SPH method is improved to realize the simulation of mesoscopic fracture of concrete. Finally, based on the proposed SPH method, the fracture process of the basalt fiber-reinforced concrete model under uniaxial compression is simulated, and the peak strength change and final fracture mode of basalt fiber-reinforced concrete are obtained. The research results provide a certain reference for the fracture mechanism and optimized design of basalt fiber-reinforced concrete.

## 2. Sample Preparation and Test Scheme

### 2.1. Sample Preparation

**Figure 2:**
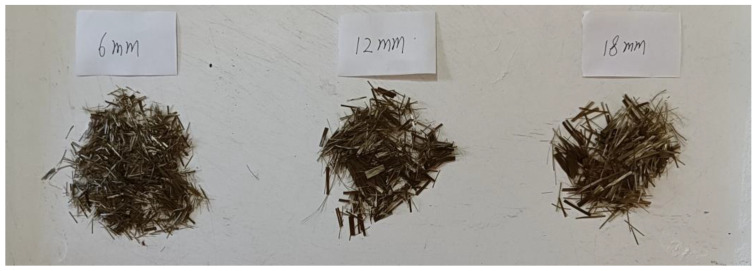
Basalt fibers of different lengths.

As there is no specific standard for basalt fiber-reinforced concrete, the preparation and curing of test blocks are carried out in accordance with the provisions in the standard SL/T352-2020 “Test Code for Hydraulic Concrete” [42]. The specific steps are as follows:

(1) Weigh cement, water, sand, gravel, water-reducing agent, and basalt fiber according to the calculated mix proportion design. The mix proportion of concrete per cubic meter is shown in Table 1 (where schemes A, B1–B3, and C1–C3 are detailed later).

(2) Add the weighed stones, sand, and cement to the concrete mixer in turn. When no fiber is added and the fiber content is 0.1%, dry mix for 30 s (to make the basalt fiber in the concrete have good dispersibility, after many attempts, when the content is 0.2% and 0.3%, the dry mixing time is 60 s and 90 s). During the mixing process, evenly spread the basalt fiber into the mixing drum. After mixing evenly, add the accurately weighed water and water-reducing agent to the concrete mixing drum, and start the mixer again. The mixing time is 90 s. Basalt fibers of different lengths are shown in Figure 2.

(3) After cleaning the mold, evenly apply release agent on its surface. Pour the well-mixed concrete into the mold. Then, fix the mold on the vibrating table and vibrate for 30 s. Subsequently, scrape off the part exceeding the test mold with a spatula and continue vibrating until the concrete volume is stable.

(4) Use a spatula to level the surface of the sample in one horizontal direction. Mark the test block with a marker pen and then put it into a standard constant temperature and humidity curing room for curing. After the curing specimen reaches 24 h, demold it, and then cure the specimen in the curing room to the specified age of 28 days.

### 2.2. Test Scheme and Test Equipment

To explore the influence of different basalt contents and single doping, as well as hybrid doping, on the mechanical properties of concrete, seven groups of test schemes are designed. Scheme A is the control group, that is, concrete without any basalt fiber, and is compared with concrete samples with different contents. Scheme B is the hybrid-doped group. The ratio of 6mm basalt fiber/12 mm basalt fiber/18 mm basalt fiber is 3:4:3. The basalt fiber contents are set as 0.1%, 0.2%, and 0.3% respectively. Scheme C is the single-doped group. A length of 18 mm basalt fiber is used. The basalt fiber contents are set as 0.1%, 0.2%, and 0.3%, respectively. The specific test schemes are shown in Table 2. The size of the sample is the standard 150 mm × 150 mm × 150 mm.

**Figure 3:**
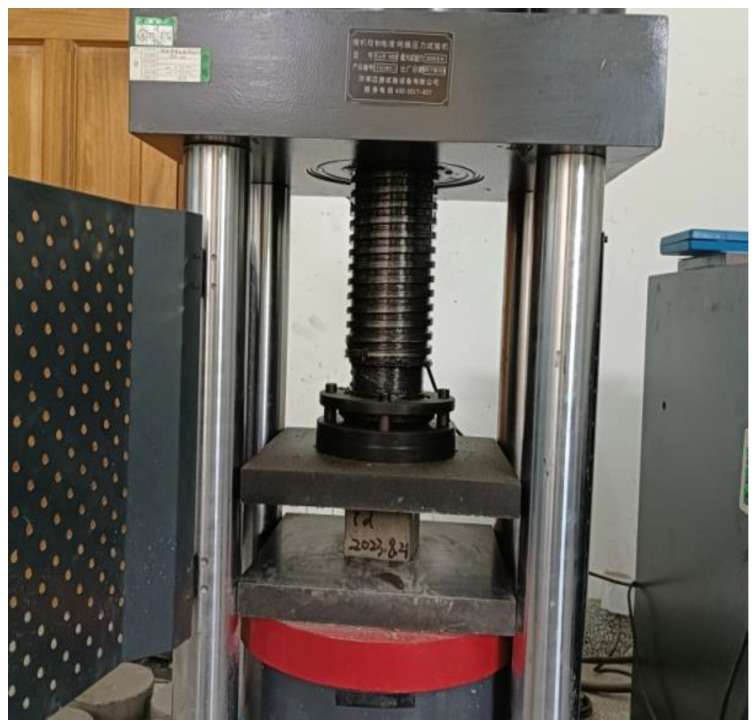
Test loading equipment.

Uniaxial compression tests on basalt fiber-reinforced concrete samples under different schemes were performed. The test equipment was a 2000 kN microcomputer servo pressure testing machine produced by Jinan Maijie Testing Equipment Co., Ltd. (Jinan, China). The model of the testing machine was YAW-2000, as shown in Figure 3. The loading mode of the testing machine was stress loading mode, and the loading rate was 5 kN/s until the sample was damaged. The peak strength and final fracture mode of the sample were recorded after loading was completed.

### 2.3. Tabular Summary of the Test Results

The analysis of the text results includes the crack morphologies, peak strength, as well as the fracture mechanisms. The detailed summary can be seen in Table 3.

## 3. Improvement of the SPH Method

### 3.1. Basic Equations of SPH

The core of SPH lies in two parts: (1) the kernel function approximation method and (2) the particle approximation method. The kernel function approximation method approximates continuous field quantity functions through discrete particles, while the particle approximation method further approximates the kernel approximation equation by applying discrete particles. The expression of the kernel function approximation method is as follows [43]:

(1) $$f(x)=\int_{\Omega }f(x^{\prime })\delta (x-x^{\prime })dx^{\prime },$$

where *x* is the particle coordinate vector; *f* represents the field quantity function and is used to represent variables such as density and velocity; *Ω* is the calculation domain of SPH; *δ* represents the Dirac function.

In SPH, the smooth kernel function *W* is generally used to replace the Dirac function. Therefore, the approximate expression of the kernel function approximation method is:

(2) $$f(x)\approx \int_{\Omega }f(x^{\prime })W(x-x^{\prime },\;h)dx^{\prime },$$

In SPH, the particle approximation method can be written as:

(3) $$f(x_{i})=\sum_{j=1}^{N}\frac{m_{j}}{\rho_{j}}f(x_{j})\cdot W_{ij},$$

In SPH, the continuity equation and momentum equation are usually used to describe the basic motion and mechanical characteristics of SPH particles. The continuity equation and momentum equation can also be collectively called the control equation, and this expression can be written as:

(4) $$\frac{d\rho_{i}}{dt}=\sum_{j=1}^{N}m_{j}v_{ij}^{\beta }\partial W_{ij,\beta },$$

(5) $$\frac{dv_{i}^{\alpha }}{dt}=\sum_{j=1}^{N}m_{j}(\frac{\sigma_{ij}^{\alpha \beta }}{\rho_{i}^{2}}+\frac{\sigma_{ij}^{\alpha \beta }}{\rho_{j}^{2}}+T_{ij})\partial W_{ij,\beta },$$

where *ρ* and *m* are the density and mass of the particle, respectively; *v* and *σ* are the velocity and stress of the particle, respectively; the subscripts *i* and *j* of the parameters represent the particle serial numbers, respectively; *t* represents the time parameter; *N* is the total number of SPH particles; *α* and *β* are Einstein notations.

### 3.2. Random Fiber Generation Algorithm Under SPH Framework

The generation of random fibers involves the generation of random numbers. In this section, the Monte Carlo method is used to generate random numbers. The method for generating the random numbers is the linear congruence method. Its general recurrence formula is as follows:

(6) $$\left\{\begin{array}{c} x_{n}=(ax_{n-1}+c)(\mathrm{mod}M)\\ r_{n}=\frac{x_{n}}{M}\\ \;\mathrm{Initial}\;\;\mathrm{value}\;\;x_{0} \end{array}\right.$$

In Equation (6), *M* is the modulus; mod *M* represents taking the remainder of the modulus; *a* is the multiplier; *c* is the increment; *x*_0_ is the initial value; *r_n_* is a random number uniformly distributed in the interval (0,1).

The distribution of basalt fiber can be equivalent to a line segment in two-dimensional space. It has three characteristics: center point position, direction angle, and length. By determining the above three parameters, the specific position of each basalt fiber in space can be determined. This paper compiles the corresponding Fortran computer program. The specific generation steps are as follows:

(1) Determine the sizes of the generation domain and the analysis domain.

The generation domain of basalt fiber is the peripheral area that contains all basalt fibers, and the analysis domain is the area involved in the calculation. For a two-dimensional model, the analysis domain is the size of the basalt concrete model, 150 mm × 150 mm. Considering the integrity of basalt fiber generation, the generation domain of basalt fiber is 18 mm inward from the analysis domain of 150 mm × 150 mm, that is, 132 mm × 132 mm.

(2) Determine the basalt fiber parameters in each group of schemes.

First, according to the generation domain determined in step (1) and the determined number of basalt fibers *n*, then randomly generate *n* random numbers within the range of (0,1). Firstly, determine the center point of the basalt fiber as (*x*_0_, *y*_0_), which is generated according to random numbers; the length of the basalt fiber is determined according to the actual scheme and defined as *l*; the direction angle is *θ*, which is randomly generated. Therefore, the endpoint coordinates of the basalt fiber are:

(7) $$x=x_{0}+(l/2)\cos\theta ,$$

(8) $$y=y_{0}+(l/2)\sin\theta .$$

Through the above method, the endpoint coordinates of *n* basalt fibers can be obtained and stored in an array.

(3) Generate a random basalt fiber model using SPH.

Based on the endpoint coordinates of each random basalt fiber obtained in step (2), import them into the SPH program. Take several uniformly distributed points on the basalt fiber line segment (where the point distance is less than the SPH particle spacing), and assign a search radius *r* (*r* > SPH particle spacing) to each point. For the SPH particles covered inside the circle with search radius *r*, they are considered as basalt fiber particles and are assigned the mechanical parameters of basalt fiber.

**Figure 4:**
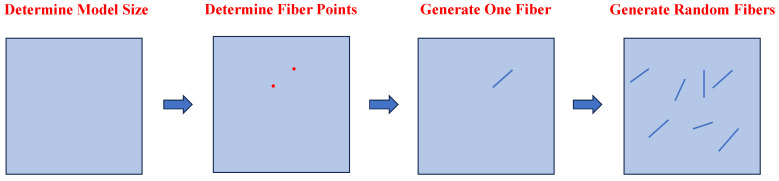
Generation process of the random fibers.

The generation process of the random fibers can be illustrated in Figure 4.

### 3.3. Numerical Treatment Method of Mesoscopic Fracture in SPH

Before performing the SPH fracture simulation, the fracture criterion needs to be determined first. In this section, the Mohr–Coulomb criterion is introduced to determine whether the SPH particles fail or not. The expression can be written as [44]:

(9) $$\sigma_{1}=\sigma_{t},$$

(10) $$\tau_{f}=c+\sigma_{f}\tan\varphi ,$$

where *σ*_1_ is the maximum principal stress on the SPH particle; *σ_t_* is the tensile strength of the SPH particle; *φ* is the particle internal friction angle in SPH; *σ_f_* and *τ_f_* are the tensile stress and shear stress on the material failure surface, respectively.

**Figure 5:**
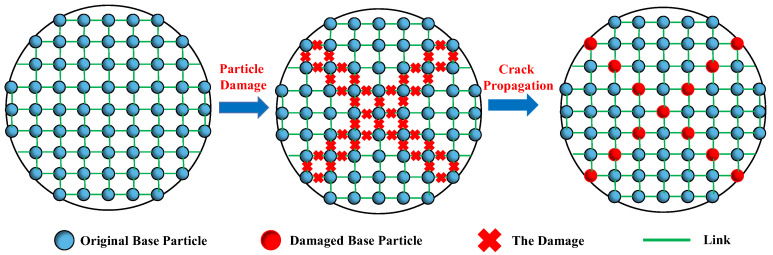
Failure process of SPH particles.

To characterize the failure of SPH particles, a failure coefficient *ξ* is introduced, and its default value is 1. When the stress on the SPH particle satisfies Equation (9) or Equation (10), the value of the failure coefficient *ξ* is set to 0, indicating that the particle is damaged. The particle failure process in SPH is shown in Figure 5. The kernel function considering particle failure characteristics is defined as *I*, while the traditional SPH kernel function is *W*. Then, the kernel function *I*, considering particle failure characteristics, can be expressed as:

(11) $$I(x-x^{\prime },\;h)=\xi \cdot W(x-x^{\prime },\;h).$$

Using the kernel function *I*, considering the particle failure process to replace the traditional kernel function *W*, the SPH control equation, considering particle failure characteristics, can be rewritten as:

(12) $$\frac{d\rho_{i}}{dt}=\sum_{j=1}^{N}m_{j}v_{ij}^{\beta }\partial I_{ij,\beta },$$

(13) $$\frac{dv_{i}^{\alpha }}{dt}=\sum_{j=1}^{N}m_{j}(\frac{\sigma_{ij}^{\alpha \beta }}{\rho_{i}^{2}}+\frac{\sigma_{ij}^{\alpha \beta }}{\rho_{j}^{2}}+T_{ij})\partial I_{ij,\beta }.$$

### 3.4. Numerical Model and Calculation Parameters

**Figure 6:**
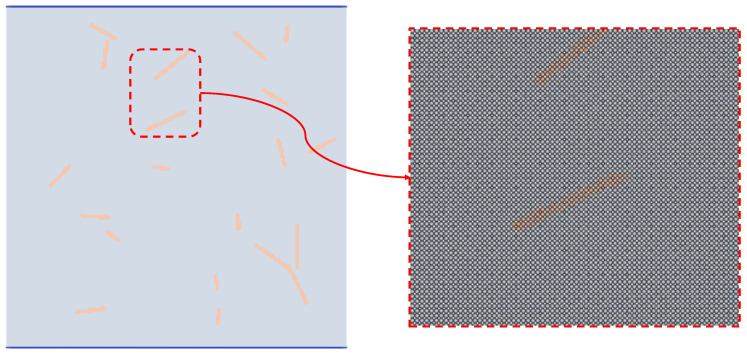
Basalt fiber-reinforced concrete model and particle subdivisions.

Figure 6 shows the numerical model of basalt fiber-reinforced concrete (taking scheme B2 as an example) and the schematic diagram of particle subdivision. The model size is consistent with the test size, which is 150 mm × 150 mm. The entire model is divided into 200 × 200 = 40,000 particles. The upper and lower parts of the model are loading boundaries (the blue part in Figure 5). The material parameters of the model are as follows: (1) Concrete matrix: Elastic modulus *E_b_* = 28 GPa, tensile strength *σ_t_* = 1 MPa, cohesion *c* = 5.95 MPa, internal friction angle *φ* = 40°, Poisson’s ratio *μ* = 0.2; (2). Basalt fiber: Elastic modulus *E_f_* = 90 GPa, tensile strength *σ_t_* = 2500 MPa, cohesion *c* = 5.95 MPa, internal friction angle *φ* = 40°, Poisson’s ratio *μ* = 0.2.

## 4. Analysis of Test and Numerical Simulation Results

### 4.1. Fracture Morphologies of Fiber-Reinforced Concrete Under Different Schemes

**Figure 7:**
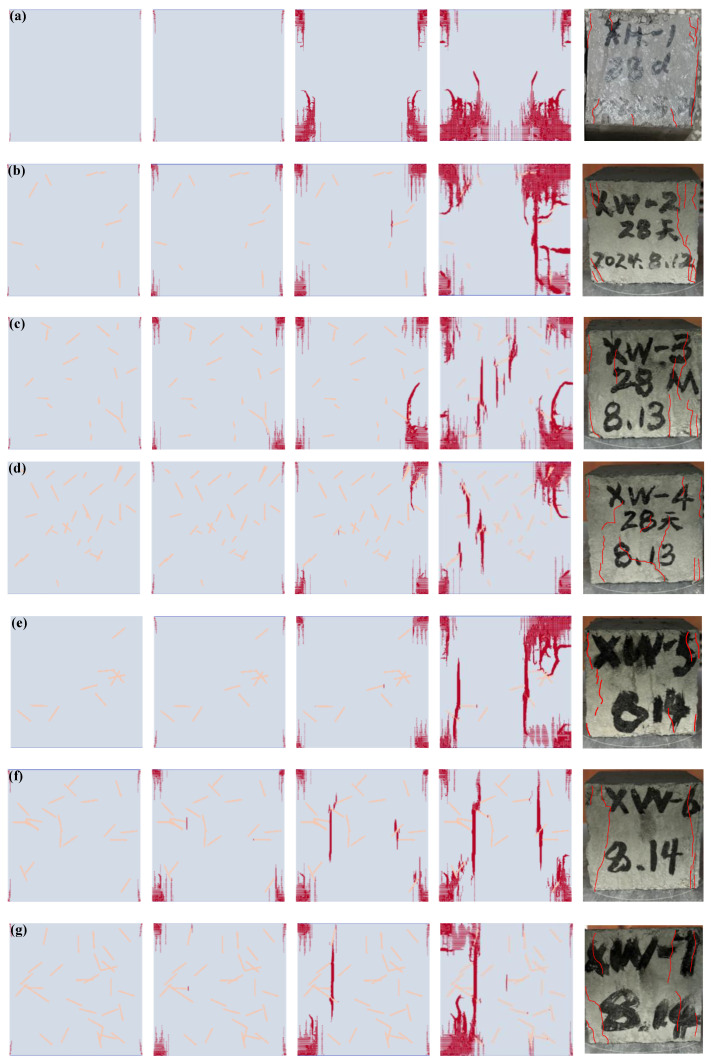
Numerical simulation results of the fracture process and the final test failure morphology of basalt fiber-reinforced concrete under different schemes. (**a**) Scheme A; (**b**) Scheme B1; (**c**) Scheme B2; (**d**) Scheme B3; (**e**) Scheme C1; (**f**) Scheme C2; (**g**) Scheme C3.

Figure 7 presents the numerical simulation outcomes of the fracture process and the final test failure morphology of basalt fiber-reinforced concrete under diverse schemes. The initial four figures depict the fracture process of basalt fiber-reinforced concrete in the numerical simulation, while the last figure shows the final failure morphology in the test. It is observable from the figure that, regarding the final failure morphology of the test, in the absence of basalt fiber, when the specimen is compressed to the ultimate failure state, the damage predominantly occurs on the upper and lower surfaces and the side edges, yet the interior of the specimen center remains essentially intact. This indicates that significant stress concentration takes place on the upper and lower surfaces during specimen compression. Upon adding basalt fiber, longitudinal cracks commence to emerge inside the specimen. With an increase in fiber content, the crack location progressively shifts from the edge towards the middle, and the number of cracks gradually rises. This implies that a suitable increase in the fiber content within concrete might enhance the stress state of the concrete, transform eccentric compression into axial compression, and indirectly augment the compressive strength of the concrete.

Further examination reveals that the addition of fibers is advantageous for ameliorating the stress concentration sites within the concrete. The fiber fully utilizes its strength advantage. Through the bonding force with the mortar interface, the mortar on both sides of the crack is interconnected. The fiber filaments can transmit pressure, enabling all parts of the structure to jointly bear the force, thereby fortifying the toughness of the concrete. Moreover, in the absence of fiber addition, the specimen exhibits brittle failure as a whole. As the fiber content escalates, the toughness of the concrete gradually augments. When ultimate failure is attained, conspicuous macroscopic phenomena such as crack propagation are evident, which facilitates the timely detection of structural failure signs and enhances the safety of the project.

From the numerical simulation results, under the circumstance of the control group scheme A, for concrete without basalt fiber, cracks first occur at the four corners of the specimen, which are shear cracks. As the load continues to increase, the shear cracks continue to develop and gradually gather in the middle of the specimen. At the same time, more cracks are generated on the surface of the specimen. Crack propagation occurs around and on the surface of the specimen in the test, which is consistent with the numerical simulation results and verifies the rationality of the numerical simulation. For scheme B with mixed basalt fibers, when the mixing percentage is 0.1% (scheme B1), shear cracks first occur around the model. Then, cracks initiate at a fiber inside the model and then expand in the vertical direction, eventually leading to splitting failure of the model. When the mixing percentage is 0.2% (scheme B2), the shear cracks are consistent with scheme B1 and first occur around the model. Due to the increase in the number of basalt fibers at this time, cracks occur at two fibers and expand in the vertical direction. Finally, the failure mode of the model is splitting failure. When the mixing percentage is 0.3% (scheme B3), shear cracks first occur around the model, and then tensile cracks occur at the basalt fibers and expand along the loading direction, resulting in splitting failure of the model. From the final failure morphology of the test specimen, in addition to the shear failure generated around the model, the concrete specimen also has splitting failure in the middle of the specimen. At the same time, as the content of basalt fiber increases, the splitting failure in the middle of the specimen also gradually increases, indicating that the basalt fiber has fully exerted its effect, and the numerical simulation results are consistent with the test results. For scheme C with a single basalt fiber, its failure mode is similar to that of scheme B. However, the difference is that due to the same length of basalt fiber in single doping which are all 18 mm, the number of splitting failures in the middle of the specimen is significantly reduced, indicating that the basalt fiber has not fully exerted its effect, so the specimen is more easily damaged.

### 4.2. Variation Law of Peak Strength of Basalt Fiber-Reinforced Concrete Specimens

**Figure 8:**
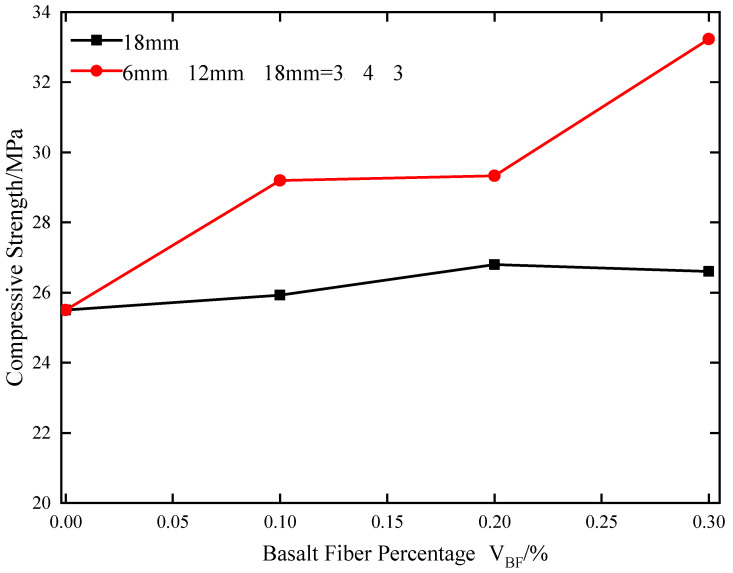
Variation laws of uniaxial peak strength of basalt fiber-reinforced concrete under different schemes.

Figure 8 shows the variation law of uniaxial peak strength of basalt fiber-reinforced concrete under different schemes. As can be seen from the figure, the strength of concrete specimens with basalt fiber added is significantly improved compared to the strength of concrete specimens without the basalt fiber. For concrete with single basalt fiber addition, the strength of concrete specimens with basalt content of 0.1%, 0.2%, and 0.3% is increased by 1.69%, 5.10%, and 4.31%, respectively, compared to the strength of concrete specimens without basalt fiber. It can be seen that with the increase in single basalt fiber content, the concrete strength is improved to a certain extent, but the improvement degree is not large. For concrete with mixed basalt fiber addition, the strength of concrete specimens with basalt content of 0.1%, 0.2%, and 0.3% is increased by 14.51%, 15.02%, and 30.31%, respectively, compared to the strength of concrete specimens without basalt fiber. Therefore, compared to the single basalt fiber addition process, mixed addition is easier to improve the strength of concrete.

## 5. Discussion

### 5.1. Analysis of Uniaxial Compression Fracture Mechanism of Basalt Fiber-Reinforced Concrete

**Figure 9:**
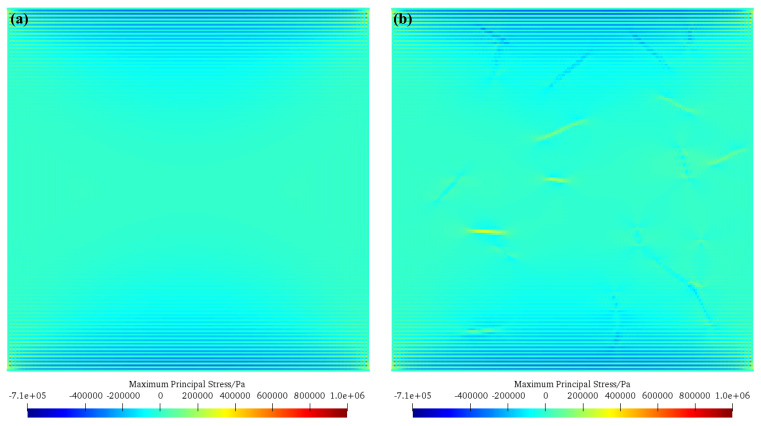
Maximum principal stress nephogram of concrete without basalt fiber (scheme A) and concrete with basalt fiber (scheme C2) before crack initiation. (**a**) Concrete without basalt fiber (scheme A); (**b**) concrete with basalt fiber (scheme C2).

Figure 9 shows the maximum principal stress nephogram of concrete without basalt fiber (scheme A) and concrete with basalt fiber (scheme B2) before the crack initiation. As can be seen from the figure, in the concrete model without basalt fiber, obvious compressive stress concentration occurs at the end of the model, and a smaller compressive stress distribution appears in the middle of the model. For the concrete model with basalt fiber, a small tensile stress appears at the basalt fiber in the middle of the model. Due to the large tensile strength of basalt fiber, the internal stress of concrete can be adjusted through basalt fiber, which can better improve the internal stress characteristics of concrete and improve the strength and toughness of concrete.

**Figure 10:**
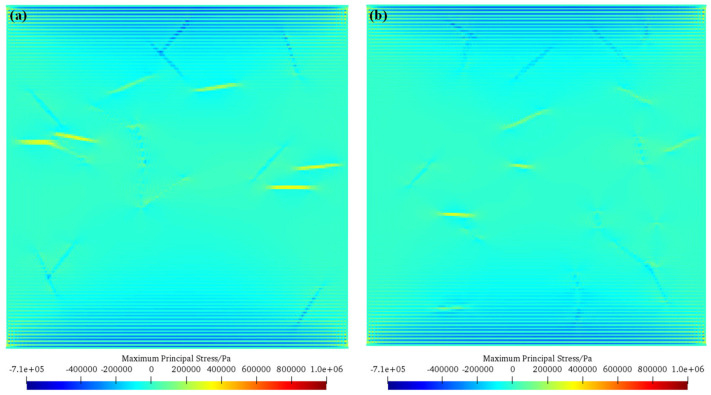
Maximum principal stress nephogram of mixed basalt fiber-reinforced concrete (scheme B2) and single-doped basalt fiber-reinforced concrete (scheme C2) before crack initiation. (**a**) Hybrid-doped basalt fiber-reinforced concrete (scheme B2); (**b**) single-doped basalt fiber-reinforced concrete (scheme C2).

Figure 10 shows the maximum principal stress nephogram of mixed basalt fiber-reinforced concrete (scheme B2) and single basalt fiber-reinforced concrete (scheme C2) before crack initiation. As can be seen from the figure, for the mixed basalt fiber-reinforced concrete, compared to the single basalt fiber-reinforced concrete model, due to the different lengths of basalt fibers inside, the tensile stress concentration at the fibers inside is not large. Therefore, under the same conditions, the mixed basalt fiber-reinforced concrete model can bear a greater load and thus has greater strength.

### 5.2. Application Prospects of the SPH Method in Fracture Simulation of Basalt Fiber-Reinforced Concrete

In this paper, uniaxial compression tests of basalt fiber-reinforced concrete were carried out. Based on the traditional SPH method, a meshless numerical method that can simulate the progressive failure process of basalt fiber-reinforced concrete was developed. Compared to the finite element method, SPH overcomes the problem of mesh redivision in dealing with large deformation and discontinuous problems. Compared to the discrete element method, the calculation parameters in SPH are strictly derived from partial differential equations, which have clear physical meanings and do not require complex parameter calibration. Therefore, SPH has broad application prospects in the failure simulation of basalt fiber-reinforced concrete.

However, the failure of real basalt fiber-reinforced concrete is a complex three-dimensional problem, but in this paper, it is only abstracted as a two-dimensional problem. The calculation of three-dimensional SPH requires a large amount of computing resources. In addition, basalt fiber-reinforced concrete has a complex meso-structure inside. At present, some scholars have used CT scanning to obtain a complex three-dimensional basalt fiber-reinforced concrete reconstruction model. Therefore, future research directions should focus on developing high-performance parallel meshless programs and taking the meso-structure of three-dimensional basalt fiber-reinforced concrete into consideration.

## 6. Conclusions

(1) Concrete tests with basalt fiber were prepared. Uniaxial compression tests of single-doped and mixed-doped basalt fiber-reinforced concrete were carried out. The failure mode and variation law of tensile strength of basalt fiber-reinforced concrete were obtained.

(2) The smooth kernel function was improved under the traditional SPH framework to realize the simulation of SPH particle failure and destruction. Embedding the generation method of random basalt fiber into the SPH algorithm can realize the generation of random basalt fiber in the basalt fiber-reinforced concrete model.

(3) When no basalt fiber is added, when the specimen is compressed to the ultimate failure state, the damage mainly occurs on the compression surface and the side edge side, while the interior of the specimen center is basically intact, indicating that obvious stress concentration occurs on the compression surface when the specimen is compressed. When basalt fiber is added, longitudinal cracks begin to appear inside the specimen, and as the content increases, the crack location gradually develops from the edge to the middle, and the number gradually increases, indicating that appropriately increasing the fiber content in concrete may improve the stress state of concrete, change the eccentric compression to axial compression, and indirectly increase the compressive strength of concrete.

(4) Simulation results are consistent with the test results, which verifies the rationality of the numerical simulation algorithm. For the concrete model without the basalt fiber, shear cracks occur around the model. For the concrete model with basalt fiber, in addition to shear cracks, the tensile cracks generated at the basalt fiber inside the model eventually lead to the splitting failure of the model.

(5) The strength of concrete specimens with basalt content of 0.1%, 0.2%, and 0.3% is increased by 1.69%, 5.10%, and 4.31%, respectively, compared to the strength of concrete specimens without basalt fiber. It can be seen that with the increase in single-doped basalt fiber content, the concrete strength is improved to a certain extent, but the improvement degree is not large. For concrete with mixed basalt fiber, the strength of concrete specimens with basalt content of 0.1%, 0.2%, and 0.3% is increased by 14.51%, 15.02%, and 30.31%, respectively, compared to the strength of concrete specimens without the basalt fiber. Therefore, compared to the single-doped basalt fiber process, mixed doping is easier to improve the strength of concrete.

## Figures and Tables

**Table 1:** Mix proportion of concrete per cubic meter under different schemes.

Scheme	Water/kg	Cement/kg	Sand/kg	Aggregate/kg	6 mm BF/kg	12 mm BF/kg	18 mm BF/kg
A	182.00	330.00	964.00	964.00	0.00	0.00	0.00
B1	182.00	330.00	964.00	964.00	0.84	1.12	0.84
B2	182.00	330.00	964.00	964.00	1.68	2.24	1.68
B3	182.00	330.00	964.00	964.00	2.52	3.36	2.52
C1	182.00	330.00	964.00	964.00	0.00	0.00	2.80
C2	182.00	330.00	964.00	964.00	0.00	0.00	5.60
C3	182.00	330.00	964.00	964.00	0.00	0.00	8.40

**Table 2:** Test schemes of basalt fiber-reinforced concrete.

Scheme	BF Percentage	6 mm BF/12 mm BF/18 mm BF
A	0	-----
B1	0.1%	3:4:3
B2	0.2%	3:4:3
B3	0.3%	3:4:3
C1	0.1%	-----
C2	0.2%	-----
C3	0.3%	-----

**Table 3:** Tabular summary of the test results.

Scheme	Crack Morphologies	Peak Strength	Fracture Mechanisms
A	√	√	√
B1	√	√	
B2	√	√	√
B3	√	√	
C1	√	√	
C2	√	√	√
C3	√	√	

## Data Availability

The original contributions presented in the study are included in the article, further inquiries can be directed to the corresponding author.
